# Abnormal Fibrinogen Level as a Prognostic Indicator in Coronavirus Disease Patients: A Retrospective Cohort Study

**DOI:** 10.3389/fmed.2021.687220

**Published:** 2021-06-14

**Authors:** Wei Long, Jie Yang, Zhengwei Li, Jinpeng Li, Sichao Chen, Danyang Chen, Shipei Wang, Qianqian Li, Di Hu, Jianglong Huang, Wen Zeng, Liang Guo, Xiaohui Wu

**Affiliations:** ^1^Department of Plastic Surgery, Zhongnan Hospital of Wuhan University, Wuhan, China; ^2^Department of Thoracic Surgery, Shanghai Pulmonary Hospital, Tongji University School of Medicine, Shanghai, China; ^3^Department of Neurosurgery, Zhongnan Hospital of Wuhan University, Wuhan, China; ^4^Department of Thyroid and Breast Surgery, Zhongnan Hospital of Wuhan University, Wuhan, China; ^5^Department of Ophthalmology, Zhongnan Hospital of Wuhan University, Wuhan, China

**Keywords:** fibrinogen, COVID-19, prognostic indicators, clinical management, retrospective study

## Abstract

**Purpose:** The coronavirus disease (COVID-19) pandemic poses a global threat, and identification of its prognostic biomarkers could prove invaluable. Fibrinogen (FIB) could be one such indicator as coagulation and fibrinolysis abnormalities are common among COVID-19 patients. We examined the role of FIB levels in the prognosis of COVID-19.

**Methods:** This retrospective cohort study enrolled 1,643 COVID-19 patients from the Leishenshan Hospital in Wuhan, China. The follow-up was conducted from February 8, 2020 to April 15, 2020. The cohort was divided into three groups according to the FIB level on admission, and associations with mortality and disease severity were determined using Cox and logistic regression analyses, respectively. Further, Kaplan–Meier (K–M) analyses by log-rank tests were used to assess the survival of patients with varying FIB levels.

**Results:** Patients with FIB < 2.2 g/L [hazard ratio (HR): 9.02, 95% confidence interval (CI): 1.91–42.59, *P* = 0.006] and >4.2 g/L (HR: 4.79, 95% CI: 1.14–20.20, *P* = 0.033) showed higher mortality risks compared to those with FIB between 2.2 and 4.2 g/L. The survival curves showed similar results in K–M analyses (*P* < 0.001). Additionally, an elevated FIB level was associated with a greater risk of developing critical disease (odds ratio: 2.16, 95% CI: 1.04–4.46, *P* = 0.038) than a FIB level within the normal range.

**Conclusion:** Abnormal FIB levels may be associated with mortality risk among COVID-19 patients and could predict critical disease development. Thus, assessment of FIB levels may assist in determining the prognosis of COVID-19 patients.

## Introduction

The coronavirus disease (COVID-19), caused by severe acute respiratory syndrome coronavirus 2 (SARS-CoV-2), was first reported in Wuhan, China in December 2019 and has since become a pandemic (1–4). As of March 26, 2021, confirmed COVID-19 cases were reported in over 200 countries and regions, with over 125 million infected people and over 2 million attributable deaths (5). The Leishenshan Hospital in Wuhan, China, was a rapidly built facility designated for COVID-19 treatment, wherein 1,880 patients were admitted and treated. The hospital undertook important medical tasks during the Wuhan outbreak and was functional from February 8, 2020 to April 15, 2020.

In recent decades, two serious epidemics have been caused by coronaviruses, namely severe acute respiratory syndrome (SARS) and Middle East respiratory syndrome (MERS) (6, 7). SARS-CoV-2 is homologous with the pathogens of SARS and MERS (8), and similarities exist between the pathologic physiology and clinical manifestations of COVID-19, SARS, and MERS. The association of SARS and MERS with thrombotic complications and coagulation manifestations suggests the importance of coagulation disturbances in COVID-19 patients (9, 10). Although most COVID-19 infections are mild and self-healing, some can cause serious complications such as acute respiratory distress syndrome (ARDS), shock, multiple system organ failure, and even death (11–13). Hence, researching factors related to potential deterioration in such cases is of great importance.

Hyperfibrinolysis is proposed to exist in complicated diseases, and abnormal coagulation indexes have been detected in COVID-19 patients (14). Fibrinogen (FIB) is a significant blood coagulation factor composed of a fibrous glycoprotein and three pairs of polypeptide chains. Also known as factor I, it is involved in fibrin gel formation during the final phase of the coagulation process (15, 16). However, evidence of the role of FIB in COVID-19 cases is lacking. Therefore, we aimed to explore the relationship between FIB levels and the prognoses of COVID-19 patients.

## Patients and Methods

### Study Design and Participants

This was a retrospective, single center study conducted in the Leishenshan Hospital. Of 1,880 RT-PCR-confirmed patients admitted to the facility, those without data on FIB levels were excluded and the remainder 1,643 were enrolled in the cohort. The follow-up period lasted until April 15, 2020.

### Ethical Approval

This retrospective study was approved by the Research Ethics Commission of Zhongnan Hospital of Wuhan University (No. 2020074), and considering the rapid spread of COVID-19, the requirement for patient consent was waived by the ethics commission.

### Data Collection

All information was obtained from the electronic medical record system, including demographic and clinical features, laboratory test results, imaging findings, treatment, and outcomes. The information was then aggregated and typed in a pre-designed format. The data were independently collected by two investigators and further examined for accuracy by two others.

### Definitions

**Figure 1:**
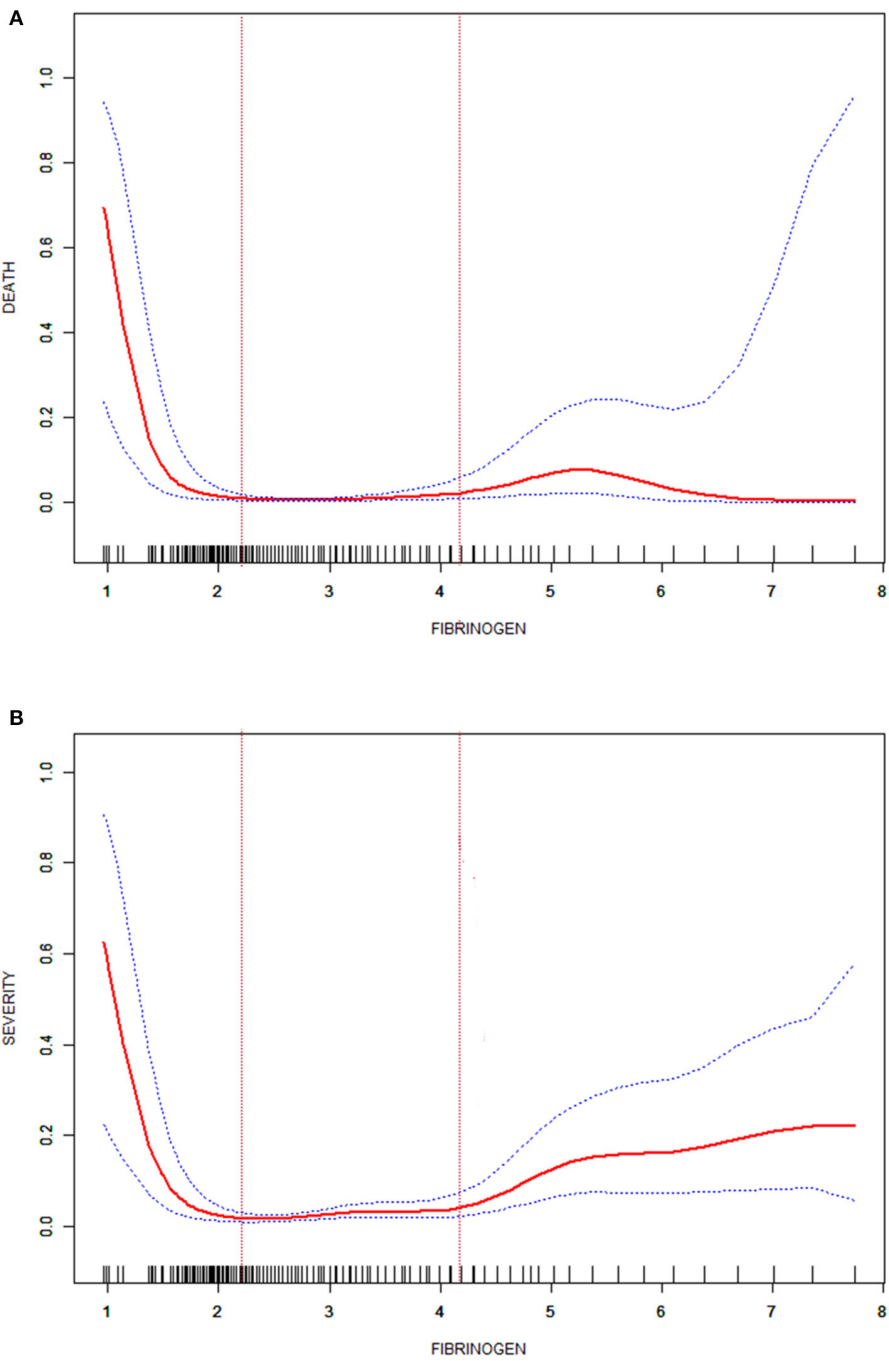
Fitted curve of the fibrinogen level and death in patients with COVID-19 **(A)**; fitted curve of the fibrinogen level and severity in patients with COVID-19 **(B)**.

The cohort was divided into three groups according to the FIB level on admission. The cut-off points (2.2, 4.2) were obtained by the curve fitting analyses of FIB levels and death or critical disease (Figure 1). In the analyses, survival (alive or dead) and disease severity were regarded as the primary outcomes; death and critical disease were coded as 1, while no death and non-critical disease were coded as 0. The seventh version of the guidelines for diagnosis and treatment of COVID-19, published by the National Health Commission, divides disease severity into mild, common, severe, and critical.

Imaging findings were also considered a vital outcome measure in this study. Two radiologists evaluated the chest computerized tomography (CT) images of all enrolled patients and allotted scores to each image after discussion. Ground-glass opacities (GGO) and reticulation or cord changes were generally observed on chest CT images in the early stage. Consolidation and pleural effusion, the primary manifestations present in the advanced stage, were rarely seen (17). In our CT scoring system, these four manifestations were assigned 1 point each and their aggregate was termed Score 1. Score 2 reflected the area of pulmonary involvement: no involvement = 0 point; <25% of pulmonary involvement = 1 point; 26–50% of pulmonary involvement = 2 points; 51–75% of pulmonary involvement = 3 points; and more than 75% of pulmonary involvement = 4 points. The sum of Score 1 and Score 2 was called the total score. The number of days was calculated using the date of onset and the date of CT scan.

### Statistical Analyses

Median [interquartile range (IQR)] and frequency (percentage) were used to express continuous and categorical variables, respectively. Kruskal–Wallis tests for continuous variables and chi-square tests for categorical variables were conducted to discern the differences in baseline characteristics among the three groups. Further, while univariate and multivariate Cox regression analyses were adopted to explore the impact of FIB on survival, univariate and multivariate logistic regression analyses were used to investigate the association between disease severity and FIB. Survival of COVID-19 patients with varying FIB levels was evaluated by Kaplan–Meier analyses using log-rank tests. Finally, curve fitting analyses were used to examine the CT scores over time and at the cut-off points. Moreover, the correlations between FIB and thrombin time (TT) and between FIB and D-dimer were expressed by fitted curves. All statistical analyses were performed using SPSS, version 22.0 (IBM Corp., Armonk, NY, USA) and EmpowerStats, version 2.0, (X&Y Solutions Corp., Boston, MA, USA). A two-sided *P* < 0.05 was considered statistically significant.

## Results

### Participant Baseline Characteristics

This study cohort of 1,643 patients was distributed as follows: 1,246 patients (median age: 59 years, IQR: 49–68 years) with FIB between 2.2 and 4.2 g/L comprised one group; 188 patients (median age: 52 years, IQR: 38–63 years) with FIB levels <2.2 g/L comprised the second group; and 209 patients (median age: 64 years, IQR: 55–72 years) with FIB levels >4.2 g/L comprised the third group. The overall distribution of fibrinogen levels at admission in all patients was detailed shown in Supplementary Table 2.

As seen in Table 1, patient groups with FIB < 2.2 g/L and FIB > 4.2 g/L had no significant difference in symptoms on admission compared to the group with 2.2 g/L ≤ FIB ≤ 4.2 g/L (*P* > 0.05); however, the former had greater mortality and rate of critical disease than the latter (*P* < 0.001). Moreover, Table 2 illustrates that the parameters of coagulation function statistically differed among patients grouped by FIB levels (*P* < 0.001) and the proportions of normal D-dimer vs. high D-dimer levels.

**Table 1:** Demographic and clinical features of 1643 patients with COVID-19.

**Covariate**	**Total (*n* = 1,643)**	**2.2 ≤ FIB ≤ 4.2, g/L, *n* = 1,246**	**FIB < 2.2, g/L, *n* = 188**	**FIB > 4.2, g/L, *n* = 209**	***P*-value**
Age, year	59 (49–68)	59 (49–68)	52 (38–63)	64 (55–72)	<0.001
Sex					0.019
Female	861 (52.4)	673 (54.0)	97 (51.6)	91 (43.5)	
Male	782 (47.6)	573 (46.0)	91 (48.4)	118 (56.5)	
Comorbidity	490 (29.8)	348 (27.9)	36 (19.1)	106 (50.7)	<0.001
Cardiovascular disease	321 (19.5)	233 (18.7)	20 (10.6)	68 (32.5)	<0.001
Pulmonary disease	85 (5.2)	56 (4.5)	4 (2.1)	25 (12.0)	<0.001
Nervous system disease	51 (3.1)	33 (2.6)	3 (1.6)	15 (7.2)	0.001
Endocrine disease	126 (7.7)	92 (7.4)	8 (4.3)	26 (12.4)	0.395
Malignancy	60 (3.7)	43 (3.5)	4 (2.1)	13 (6.2)	0.635
Digestive system disease	41 (2.5)	27 (2.2)	7 (3.7)	7 (3.3)	0.310
Severity of COVID-19 when admission					<0.001
Mild	525 (32.7)	415 (34.1)	50 (27.0)	60 (29.9)	
Common	771 (48.0)	595 (48.8)	107 (57.8)	69 (34.3)	
Sever	284 (17.7)	195 (16.0)	24 (13.0)	65 (32.3)	
Critical	25 (1.6)	14 (1.1)	4 (2.2)	7 (3.5)	
The highest level of severity at hospitalization					<0.001
Mild	1 (0.1)	1 (0.1)	0	0	
Common	813 (49.7)	647 (52.1)	109 (58.3)	57 (27.4)	
Severe	774 (47.3)	570 (45.7)	72 (38.5)	132 (63.5)	
Critical	50 (3.0)	24 (1.9)	7 (3.7)	19 (9.1)	
The highest level of oxygen support					<0.001
Low flow oxygen therapy	248 (15.1)	178 (14.3)	37 (19.7)	33 (15.8)	
High flow oxygen therapy	40 (2.4)	23 (11.4)	0	17 (8.1)	
Tracheal intubation	5 (0.3)	1 (0.5)	3 (1.6)	1 (0.5)	
ECMO	1 (0.1)	0	0	1 (0.5)	
Symptoms when admitted to the hospital					
Fever or myalgia	625 (78.8)	457 (78.7)	56 (76.7)	112 (80.6)	0.795
Respiratory system symptoms	639 (80.6)	466 (80.2)	60 (82.2)	113 (81.3)	0.897
Digestive system symptoms	85 (10.7)	63 (10.8)	6 (8.2)	16 (11.5)	0.749
Nervous system symptoms	26 (3.3)	21 (3.6)	2 (2.7)	3 (2.2)	0.662
Other system symptoms	27 (3.4)	17 (2.9)	5 (6.8)	5 (3.6)	0.217
Antiviral therapy	770 (46.9)	567 (45.5)	88 (47.3)	114 (54.5)	0.053
Antibiotic therapy	461 (28.1)	323 (25.9)	46 (24.5)	92 (44.0)	<0.001
The appliance of vitamin C	241 (14.7)	184 (14.8)	21 (11.2)	36 (17.2)	0.230
Traditional Chinese medicine therapy	1,406 (85.6)	1,073 (86.1)	160 (85.1)	173 (82.8)	0.437
Anticoagulation treatment	134 (8.2)	76 (6.1)	13 (6.9)	45 (21.5)	<0.001
Use of corticosteroid	96 (5.8)	55 (4.4)	12 (6.4)	29 (13.9)	<0.001
Use of antimalarial	130 (7.9)	99 (7.9)	17 (9.0)	14 (6.7)	0.686
Days in hospital	18 (13–24)	18 (13–24)	15 (11–21)	15 (20–29)	<0.001
CT scores					0.948
1–4	77 (41.2)	47 (41.6)	7 (43.8)	23 (39.7)	
5–7	110 (58.8)	66 (58.4)	9 (56.3)	35 (60.3)	
ICU admission	35 (2.1)	15 (1.2)	6 (3.2)	14 (6.7)	0.058
Death	14 (0.9)	4 (0.3)	5 (2.7)	5 (2.4)	<0.001

**Table 2:** Laboratory results of 1643 patients with COVID-19.

**Covariate**	**Total (*n* = 1,643)**	**2.2 ≤ FIB ≤ 4.2, g/L, *n* = 1,246**	**FIB < 2.2, g/L, *n* = 188**	**FIB > 4.2, g/L, *n* = 209**	***P*-value**	**Reference range**
Interleukin-6, pg/mL	1.50 (1.50–4.20)	1.50 (1.50–3.49)	1.50 (1.10–2.30)	5.50 (1.90–17.10)	<0.001	0–7.00
Procalcitonin, ng/mL	0.04 (0.03–0.06)	0.04 (0.03–0.05)	0.03 (0.02–0.04)	0.06 (0.04–0.12)	<0.001	<0.05
Alanine aminotransferase, U/L	23.00 (15.00–37.00)	23.00 (15.00–37.00)	22.00 (13.00–39.00)	24.00 (16.00–39.00)	0.690	9.00–50.00
Aspartate aminotransferase, U/L	20.00 (16.00–27.00)	20.00 (16.00–27.00)	19.00 (15.00–27.00)	21.00 (15.00–30.00)	0.084	15.00–40.00
Albumin, g/L	37.70 (35.00–40.10)	37.90 (35.40–40.10)	39.00 (36.00–41.70)	34.80 (32.10–37.40)	<0.001	40.00–55.00
Creatine kinase, U/L	52.00 (36.00–75.00)	53.00 (37.00–76.00)	55.00 (42.00–76.00)	42.00 (29.00–61.80)	<0.001	18.00–198.00
Lactate dehydrogenase, U/L	185.00 (160.00–217.00)	183.00 (159.00–212.00)	172.00 (152.00–200.00)	222.50 (184.50–278.80)	<0.001	125.00–343.00
Total bilirubin, μmol/L	9.20 (7.00–12.15)	9.10 (7.00–11.70)	10.80 (7.90–14.70)	8.80 (6.20–12.20)	<0.001	5.00–21.00
Total protein, g/L	65.70 (62.00–69.50)	65.90 (62.60–69.50)	66.50 (61.70–70.10)	63.80 (59.30–67.60)	<0.001	65.00–85.00
Uricacid, μmol/L	300.00 (242.00–372.00)	304.00 (248.00–372.00)	323.00 (235.00–390.00)	274.00 (225.00–351.00)	0.001	208.00–428.00
Creatinine, μmol/L	64.30 (54.50–75.80)	63.90 (54.50–75.60)	64.30 (53.40–71.90)	65.90 (55.40–81.30)	0.046	64.00–104.00
Ureanitrogen, mmol/L	4.80 (3.90–5.80)	4.80 (3.90–5.80)	4.80 (3.90–5.70)	4.80 (3.90–6.10)	0.477	2.80–7.60
International normalized ratio	0.97 (0.93–1.02)	0.97 (0.93–1.00)	0.98 (0.93–1.03)	1.00 (0.97–1.06)	<0.001	0.80–1.30
Prothrombin time, s	11.30 (10.90–11.80)	11.30 (10.90–11.60)	11.40 (10.90–11.90)	11.60 (11.30–12.20)	<0.001	9.40–12.50
Thrombin time, s	17.60 (17.00–18.40)	17.60 (17.10–18.30)	18.60 (18.00–19.80)	16.60 (15.80–17.20)	<0.001	10.30–16.60
Activated partial thromboplastin time, s	27.20 (24.60–30.40)	26.80 (24.40–30.00)	27.30 (23.60–31.40)	29.40 (26.80–32.70)	<0.001	25.10–36.50
D-dimer, ng/mL	0.39 (0.21–0.91)	0.36 (0.21–0.82)	0.25 (0.14–0.60)	0.92 (0.47–1.99)	<0.001	0–0.50
0–0.50	952 (57.9)	758 (60.8)	136 (72.3)	58 (27.8)		
>0.50	691 (42.1)	488 (39.2)	52 (27.7)	151 (72.2)		
White blood cell count, × 10^9^/L	5.72 (4.71–6.92)	5.63 (4.68–6.77)	5.33 (4.57–6.73)	6.78 (5.52–8.15)	<0.001	3.50–9.50
Neutrophil count, × 10^9^/L	3.28 (2.53–4.28)	3.19 (2.51–4.05)	2.99 (2.28–3.86)	4.47 (3.50–5.77)	<0.001	1.80–6.30
Lymphocyte count, × 10^9^/L	1.60 (1.23–1.99)	1.64 (1.29–2.00)	1.70 (1.33–2.21)	1.28 (0.96–1.62)	<0.001	1.10–3.20
Red blood cell count, × 10^9^/L	4.11 (3.75–4.47)	4.13 (3.80–4.47)	4.17 (3.79–4.67)	3.86 (3.41–4.27)	<0.001	4.30–5.80
Hemoglobin, g/L	125.50 (115.00–137.00)	126.00 (116.00–137.00)	128.50 (116.00–140.80)	117.00 (106.00–129.50)	<0.001	130.00–175.00
Platelet count, × 10^9^/L	229.00 (186.00–279.00)	230.00 (187.50–277.50)	212.00 (171.00–256.00)	246.00 (197.00–322.50)	<0.001	125.00–350.00
IgM of SARS-CoV-2	212 (36.5)	157 (36.8)	19 (31.1)	36 (38.7)	0.618	(–)
IgG of SARS-CoV-2	505 (91.5)	380 (92.7)	46 (80.7)	79 (92.9)	0.006	(–)

### Survival Analyses

**Figure 2:**
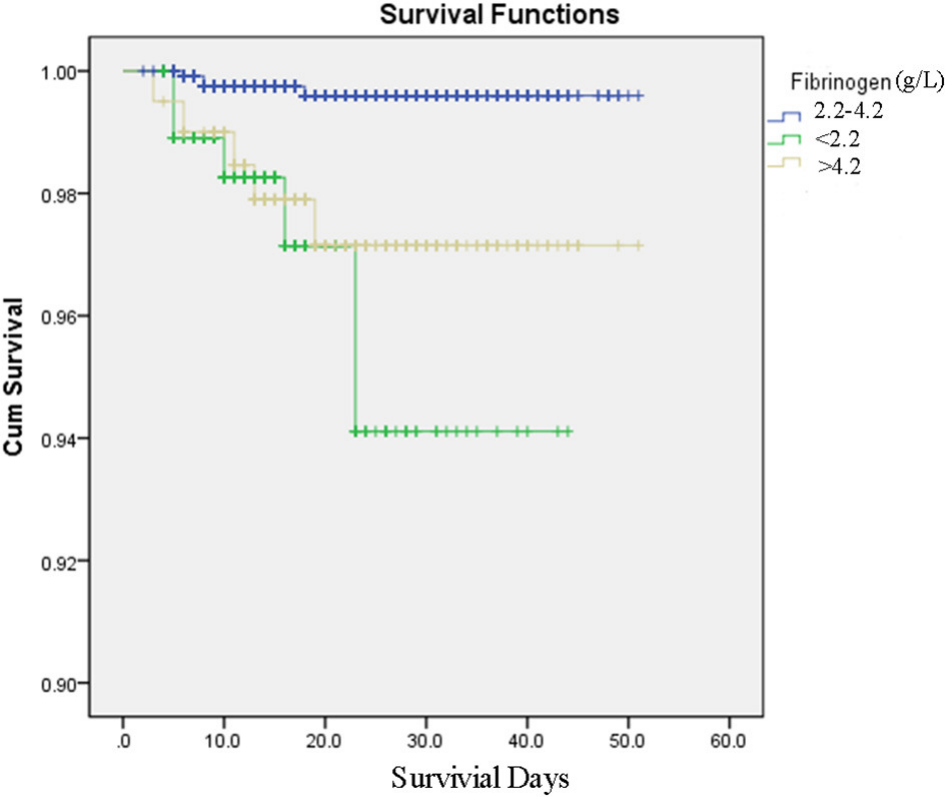
Survival curves produced by Kaplan–Meier analyses for COVID-19 patients with fibrinogen between 2.2 and 4.2 g/L, fibrinogen <2.2 g/L, fibrinogen over 4.2 g/L.

Kaplan–Meier analyses revealed that patients with FIB < 2.2 g/L or FIB > 4.2 g/L had a higher mortality risk than patients with 2.2 g/L ≤ FIB ≤ 4.2 g/L (*P* < 0.001, Figure 2). These results were consistent with those of the Cox regression analyses. The FIB levels were found to be related with COVID-19 survival in the univariate analysis. After adjusting for confounders (age, creatine kinase, total bilirubin, creatinine, white blood cell count, lymphocyte count, cardiovascular disease history), the multivariate analysis revealed that patients with FIB < 2.2 g/L [hazard ratio (HR): 9.02, 95% confidence Interval (CI): 1.91–42.59, *P* = 0.006] and FIB > 4.2 g/L (HR: 4.79, 95% CI: 1.14–20.20, *P* = 0.033) had an increased mortality risk due to COVID-19 than patients with 2.2 g/L ≤ FIB ≤ 4.2 g/L (Table 3).

**Table 3:** Univariate and multivariate Cox regression analyses for mortality in patients with COVID-19.

**Group**		**Cox regression analysis**			
		**HR**	**95 % CI**		***P-*value**
Univariate analysis	2.2 ≤ FIB ≤ 4.2, g/L	Ref			
	FIB < 2.2, g/L	9.53	2.55	35.58	0.001
	FIB > 4.2, g/L	6.97	1.87	26.00	0.004
Multivariate analysis^*^	2.2 ≤ FIB ≤ 4.2, g/L	Ref			
	FIB < 2.2, g/L	9.02	1.91	42.59	0.006
	FIB > 4.2, g/L	4.79	1.14	20.20	0.033

* *Adjusted for age, creatine kinase, total bilirubin, creatinine, white blood cell count, lymphocyte count, history of cardiovascular disease*.

### Association Between FIB and Disease Severity

Logistic regression analyses showed that the risk for critical disease in patients with FIB > 4.2 g/L [odds ratio (OR): 2.16, 95% CI: 1.04–4.46, *P* = 0.038] increased in comparison to patients with 2.2 g/L ≤ FIB ≤ 4.2 g/L; however, the risk for critical disease was not significantly different between patients with FIB < 2.2 g/L (OR: 1.81, 95% CI: 0.65–5.06, *P* = 0.258) and patients with 2.2 g/L ≤ FIB ≤ 4.2 g/L (Table 4).

**Table 4:** Univariate and multivariate logistic regression analysis for critical disease in patients with COVID-19.

**Group**		**Logistic regression analysis**			
		**HR**	**95 % CI**		***P-*value**
Univariate analysis	2.2 ≤ FIB ≤ 4.2, g/L	Ref			
	FIB < 2.2, g/L	1.96	0.83	4.62	0.123
	FIB > 4.2, g/L	5.10	2.74	9.49	<0.001
Multivariate analysis^*^	2.2 ≤ FIB ≤ 4.2, g/L	Ref			
	FIB < 2.2, g/L	1.81	0.65	5.06	0.258
	FIB > 4.2, g/L	2.16	1.04	4.46	0.038

* *Adjusted for age, creatine kinase, total bilirubin, creatinine, white blood cell count, lymphocyte count, history of cardiovascular disease*.

**Figure 3:**
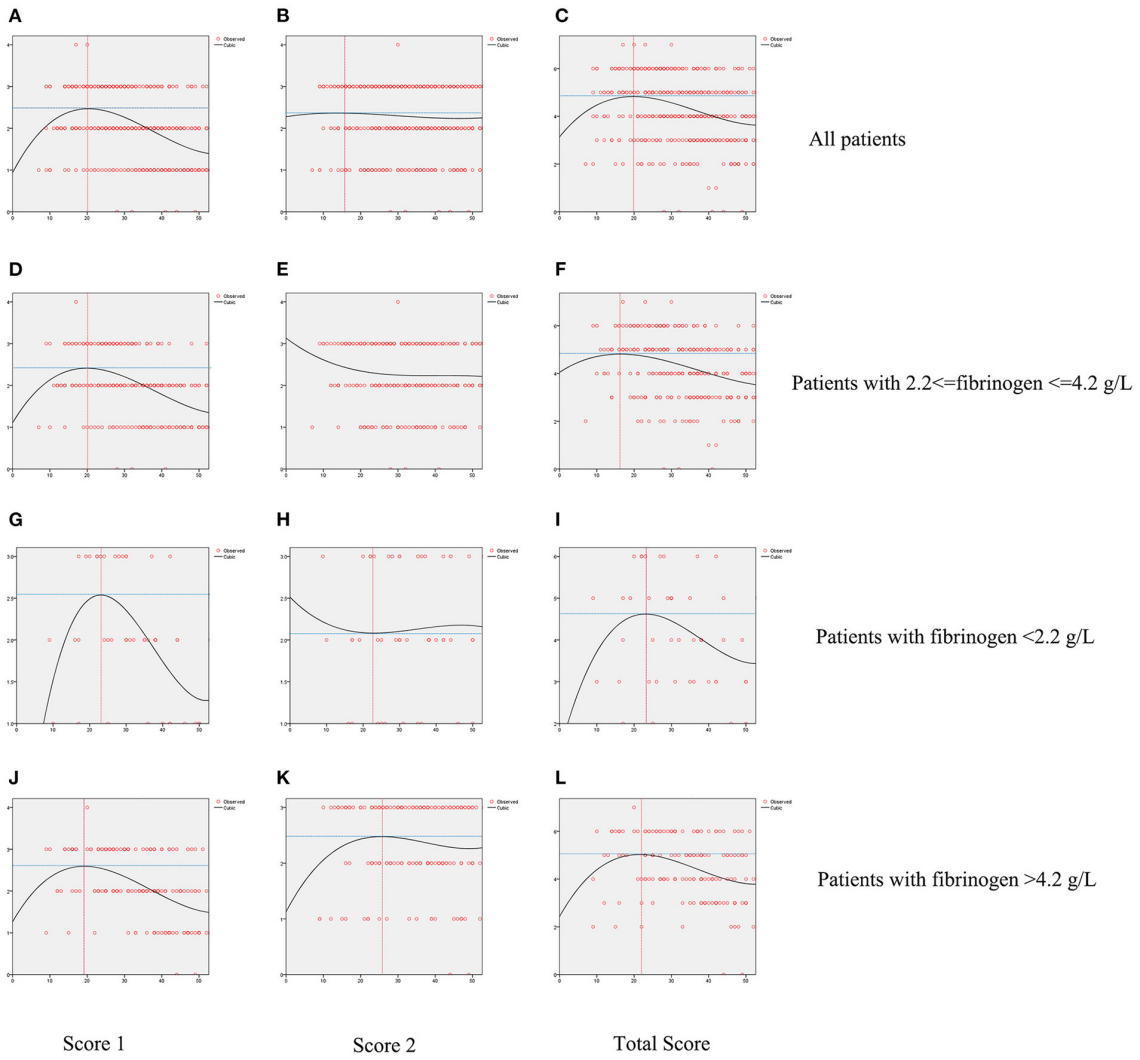
Fitted curves of patients with COVID-19 divided by levels of fibrinogen based on CT score. Dynamic changes of Score 1 **(A)**, Score 2 **(B)**, Total Score **(C)** in all patients; dynamic changes of Score 1 **(D)**, Score 2 **(E)**, Total Score **(F)** in patients with fibrinogen between 2.2 and 4.2 g/L; dynamic changes of Score 1 **(G)**, Score 2 **(H)**, Total Score **(I)** in patients with fibrinogen <2.2 g/L; dynamic changes of Score 1 **(J)**, Score 2 **(K)**, Total Score **(L)** in patients with fibrinogen over 4.2 g/L.

Figure 3 displays differences between patients by FIB levels. While Score 1 reached its peak value on ~20 days from onset for all groups, patients with FIB < 2.2 g/L or FIB > 4.2 g/L had higher peak points than others. A reduction in Score 2 was seen among patients with 2.2 g/L ≤ FIB ≤ 4.2 g/L. Contrastingly, an increase in this score appeared on day 23 among patients with FIB < 2.2 g/L and a decline appeared on day 26 for patients with FIB > 4.2 g/L. The total score of all patients showed a similar trend of initial rise and subsequent reduction. However, patients with FIB < 2.2 g/L or FIB 4.2 g/L showed a delay of inflection point.

### Association Between FIB and D-Dimer and Between FIB and TT

**Figure 4:**
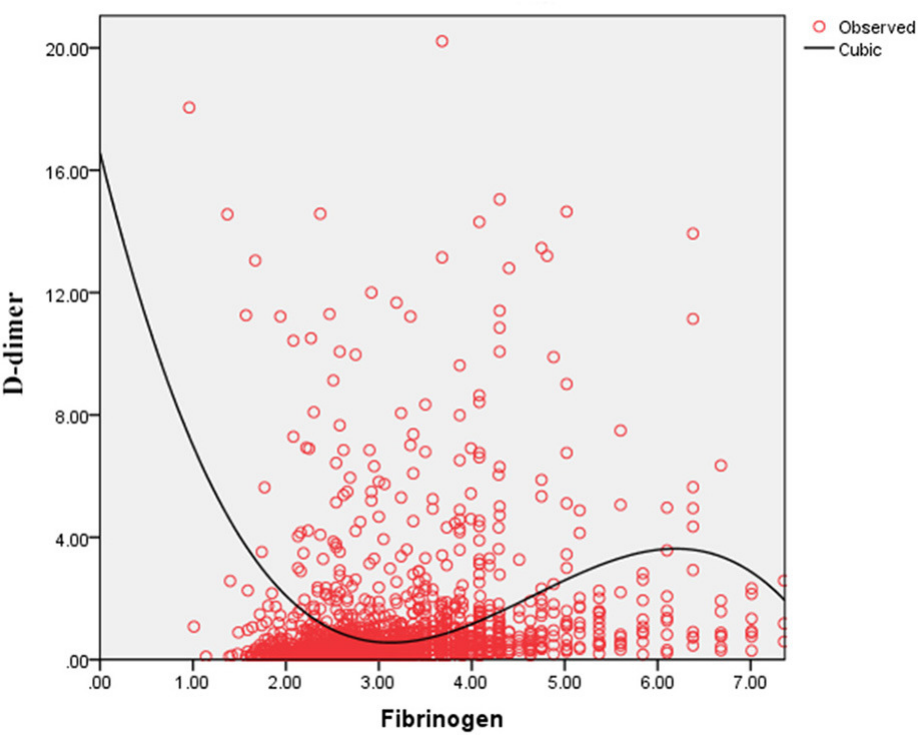
Fitted curves of the fibrinogen level and D-dimer concentration in patients with COVID-19.

**Figure 5:**
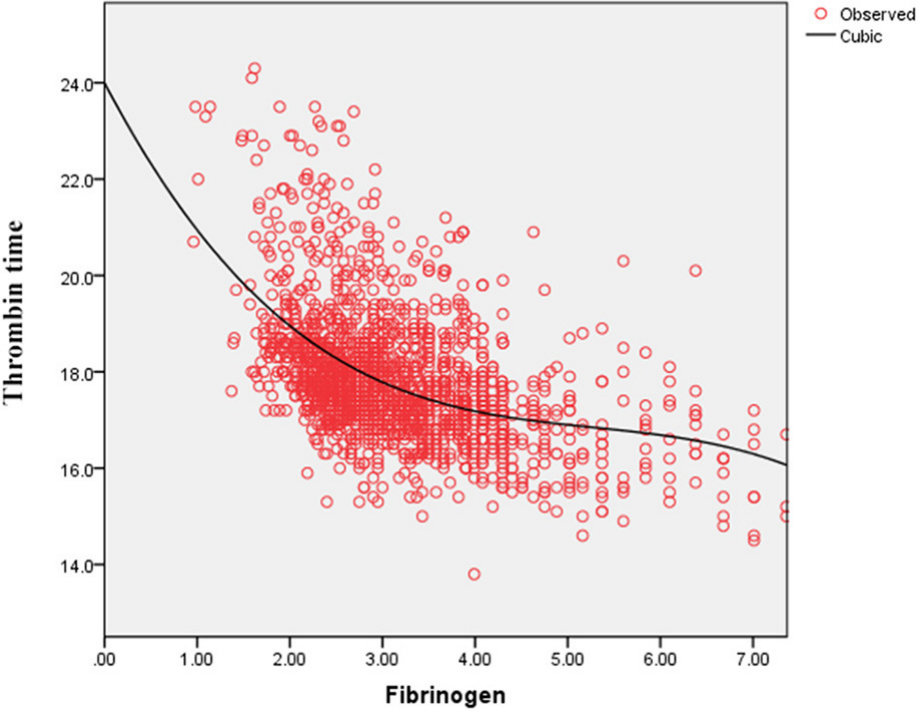
Fitted curves of the fibrinogen level and thrombin time in patients with COVID-19.

As shown in Figure 4, the serum D-dimer concentration tends to decrease initially and subsequently increase with the increase in FIB concentration. As shown in Figure 5, TT increases with decrease in the FIB level. The result suggests a negative correlation between FIB levels and TT.

## Discussion

In this retrospective cohort study, abnormal FIB levels on admission were associated with a greater risk of COVID-19-related mortality compared with normal FIB levels. Mortality risks of abnormal FIB were significantly elevated in both unadjusted and adjusted Cox analyses, and high levels of FIB indicated greater disease severity and more complications. The logistic analyses showed that elevated FIB levels were associated with a significantly greater risk of critical disease. These results indicate that FIB levels could be potential biomarkers of poor prognoses and disease deterioration in COVID-19 cases.

Thrombotic complications are likely to be treated as a vital issue in COVID-19 cases (9, 14, 18). Guan et al. reported that nearly one third of their study cohort developed thrombocytopenia (36.2%), approximately half had elevated D-dimer (46.4%), and the rate of these complications increased among patients with severe and critical disease conditions (18). Wang et al. also reported two cases of COVID-19 complicated with disseminated intravascular coagulation (DIC) (19). Additionally, Tang et al. state that 71.4% of non-survivors in their cohort met the criteria of DIC during hospitalization (20). These studies corroborate that the infection of SARS-CoV-2 is potentially accompanied by the risk of developing DIC. After infection, the damaged tissue releases tissue factors into the blood and activates the exogenous coagulation system, causing extensive microthrombus and depletion of coagulation factors (21, 22). Moreover, 27.7% of patients with FIB < 2.2 g/L owing to a high D-dimer concentration in our study and the D-dimer level correlated negatively with low FIB concentrations in the curve-fitting analysis (Figure 4). Figure 5 demonstrates that the TT increases with the decrease in FIB levels. The reduction of FIB concentrations is consistent with the hyperfibrinolysis that is associated with elevated D-dimer levels and elevated TT, which is a characteristic feature of COVID-19. The depletion of FIB and the resultant secondary hyperfibrinolysis could explain the poor prognosis of patients with low FIB levels.

In our study, patients with conditions such as old age, smoking, diabetes, heart disease, and renal insufficiency were usually in a state of hypercoagulability. Increased FIB levels on admission might indicate the concurrence of old age or comorbidities and, therefore, a poorer prognosis of COVID-19 (23, 24). Moreover, FIB is a significant positive acute-phase reactant, similar to C-reactive protein and ferritin. Positive acute-phase reactants are empirically defined as hematological components whose the plasma concentration increases following an inflammatory reaction (25, 26). During infection, the inflammatory cytokines aggregate and activate; activated inflammatory cytokines were helpful to promote the appearance of pro-coagulation state. This is consistent with the findings in our study and may explain why patients with increased FIB levels had a higher risk of mortality and critical disease severity. Acting as an acute-phase reactant, plasma FIB levels can remain within the normal range for a period, despite their ongoing consumption in DIC (27). Thus, we infer that some patients with 2.2 g/L ≤ FIB ≤ 111 4.2 g/L are in early-stage DIC and tend to develop a critical illness. This finding possibly explains why the risk of critical illness did not differ significantly between patients with FIB < 2.2 g/L and patients with 2.2 g/L ≤ FIB ≤ 4.2 g/L.

Recent autopsy studies of COVID-19 deaths indicate that bleeding and thrombotic manifestations of SARS-CoV-2 infection were critical events with a poor prognosis. One autopsy series revealed that the presence of thrombosis and associated hemorrhage significantly contributed to COVID-19 mortality. In another autopsy series of COVID-19 patients, researchers found extensive extracellular fibrin deposits and fibrin thrombi within blood vessels. The imbalance of FIB, also known as factor I, is characterized as a vital hematological manifestation of bleeding and thrombosis and is an easy parameter to measure. Therefore, we used FIB levels as the prognostic predictor of COVID-19.

Our study has several limitations. First, the study enrolled patients admitted to the Leishenshan Hospital in Wuhan, China, and therefore, the results may not be applicable to out-patients. Second, due to the retrospective nature of the study, sample heterogeneity could not be avoided. Third, we were unable to monitor the FIB levels dynamically. Last but not least, the associations of FIB concentration with the outcomes have wide CI, suggesting FIB concentration might be of limited prognostic value. So, procedures should be taken to verify the prognostic value of FIB. We calculated the sensitivity and specificity of FIB thresholds as severity prognostic indicators and compared the correlation of some clinical and laboratory factors and survival. Although all other factors compared were not better than FIB in prognosis, the sensitivity and specificity of FIB thresholds were not very high. Therefore, we recommend further research that investigates the role of FIB in determining the prognosis of COVID-19 cases through multicenter studies and larger cohorts.

In conclusion, abnormal FIB levels on admission were associated with higher risk of COVID-19-related mortality and patients with high level FIB were tended to develop a critical disease. Thus, FIB may be an effective prognostic indicator for COVID-19. This enlightens us the test of coagulation functions on admission is quite necessary and patients accompanied with abnormal FIB should be treated as high risk for deterioration.

## Data Availability Statement

The raw data supporting the conclusions of this article will be made available by the authors, without undue reservation.

## Ethics Statement

The studies involving human participants were reviewed and approved by the Research Ethics Commission of Zhongnan Hospital of Wuhan University. Written informed consent from the participants' legal guardian/next of kin was not required to participate in this study in accordance with the national legislation and the institutional requirements.

## Author Contributions

XW, LG, and WZ contributed conception and design of the study. WL performed the statistical analysis and wrote the first draft of the manuscript. JL, JY, SC, and ZL contributed to data acquisition. DC, SW, QL, DH, and JH contributed to manuscript revision. All authors have accepted responsibility for the entire content of this manuscript and approved its submission.

## Conflict of Interest

The authors declare that the research was conducted in the absence of any commercial or financial relationships that could be construed as a potential conflict of interest.

## Abbreviations

COVID-19: Coronavirus disease
CI: Confidence interval
DIC: Disseminated intravascular coagulation
FIB: Fibrinogen
HR: Hazard ratio
MERS: Middle East respiratory syndrome
SARS-CoV-2: Severe acute respiratory syndrome coronavirus 2
SARS: Severe acute respiratory syndrome
ARDS: Acute respiratory distress syndrome.
