# Supplementary material for: Abnormal Fibrinogen Level as a Prognostic Indicator in Coronavirus Disease Patients: A Retrospective Cohort Study
Source: Front Med (Lausanne). 2021 Jun 14;8:687220. doi: 10.3389/fmed.2021.687220 (PMC8236504; doi:10.3389/fmed.2021.687220)
Supplement: Supplementary file 1 [file Table_1.DOCX]

Supplement table 1: Cox regression analyses of clinical and laboratory factors for mortality in patients with COVID-19.

| Factors | Univariate Cox Regression Analysis | | | | Multivariate Cox Regression Analysis* | | | |
| --- | --- | --- | --- | --- | --- | --- | --- | --- |
|  | HR | 95 % CI | | P value | HR | 95 % CI | | P value |
| 2.2≤FIB≤4.2, g/L | ref |  |  |  | ref |  |  |  |
| FIB<2.2, g/L | 9.53 | 2.55 | 35.58 | 0.001 | 9.02 | 1.91 | 42.59 | 0.006 |
| FIB>4.2, g/L | 6.97 | 1.87 | 26.00 |  | 4.79 | 1.14 | 20.2 | 0.033 |
| Female | ref |  |  |  | ref |  |  |  |
| Male | 0.61 | 0.21 | 1.83 | 0.381 | 0.68 | 0.19 | 2.42 | 0.549 |
| Normal IL-6 | ref |  |  |  | ref |  |  |  |
| Elevated IL-6 | 29.37 | 3.53 | 243.97 | 0.002 | 4.86 | 0.43 | 55.26 | 0.203 |
| Normal PCT | ref |  |  |  | ref |  |  |  |
| Elevated PCT | 21.36 | 2.78 | 164.37 | 0.003 | 5.61 | 0.66 | 47.86 | 0.115 |
| Normal ALT | ref |  |  |  | ref |  |  |  |
| Reduced ALT | 4.21 | 0.89 | 19.89 | 0.070 | 3.82 | 0.70 | 20.82 | 0.121 |
| Elevated ALT | 2.68 | 0.81 | 8.91 | 0.107 | 1.66 | 0.37 | 7.44 | 0.507 |
| Normal UA | ref |  |  |  | ref |  |  |  |
| Reduced UA | 4.60 | 1.40 | 15.07 | 0.012 | 1.23 | 0.31 | 4.97 | 0.769 |
| Elevated UA | 3.26 | 0.81 | 13.04 | 0.095 | 0.26 | 0.03 | 2.54 | 0.248 |

*Adjusted for age, creatine kinase, total bilirubin, creatinine, white blood cell count, lymphocyte count, history of cardiovascular disease.

Supplement table 2: Overall distribution of fibrinogen levels at admission of all patients.

| **Concentration, g/L** | **Number, n=1643** |
| --- | --- |
| FIB<1.0 | 2(0.1) |
| 1.0≤FIB<1.2 | 3(0.2) |
| 1.2≤FIB<1.4 | 2(0.1) |
| 1.4≤FIB<1.6 | 9(0.5) |
| 1.6≤FIB<1.8 | 33(2.0) |
| 1.8≤FIB<2.0 | 52(3.1) |
| 2.0≤FIB<2.2 | 87(5.3) |
| 2.2≤FIB<2.4 | 128(7.7) |
| 2.4≤FIB<2.6 | 184(11.1) |
| 2.6≤FIB<2.8 | 147(8.9) |
| 2.8≤FIB<3.0 | 192(11.6) |
| 3.0≤FIB<3.2 | 128(7.7) |
| 3.2≤FIB<3.4 | 115(7.0) |
| 3.4≤FIB<3.6 | 99(6.0) |
| 3.6≤FIB<3.8 | 67(4.1) |
| 3.8≤FIB<4.0 | 106(6.4) |
| 4.0≤FIB<4.2 | 80(4.8) |
| 4.2≤FIB<4.4 | 43(2.6) |
| 4.4≤FIB<4.6 | 19(1.1) |
| 4.6≤FIB<4.8 | 32(1.9) |
| 4.8≤FIB<5.0 | 9(0.5) |
| 5.0≤FIB<5.2 | 26(1.6) |
| 5.2≤FIB<5.4 | 15(0.9) |
| FIB≥5.4 | 65(3.9) |
